# Supplementary material for: Molecular Epidemiology Analysis of SARS-CoV-2 Strains Circulating in Romania during the First Months of the Pandemic
Source: Life (Basel). 2020 Aug 14;10(8):152. doi: 10.3390/life10080152 (PMC7460100; doi:10.3390/life10080152)
Supplement: Supplementary file 1 [file life-10-00152-s001.pdf]

Supplementary Materials

Supplementary Table S1. Region-specific mutations observed in Romanian SARS-CoV-2 sequences.

**County**	**SARS-CoV-2 Genome Coding Region**							
	Orf1a	Orf1b	S	3A	7A	8	N	ORF10
Argeș	G88E		V308L				P162S	
BMA	E87D, T85I, R222C, G265V, K489E, V577F, I1683T, A1766V, T135I, Y397C	T870I, H290Y, V51A	N439K, G744S			A51V		
Buzău	T166I, S378F, L79*, P125L, V469L, T1793I	T141I, P227L, S74P, A162V		V13L, T175I				M1T
Constanța	C477Y, T327I, K618N		E96D, H245Y, P521R	K16N, S26L			P6S	
Iași	A488V, S166N, S1296T, W90*	N386S	N188K, S1242C					
Suceava	G212C, T169N	L43F, S301P, M576I	L5F, S640F		A105V			
Vâlcea		A302V, P504S			V104F		T16M	

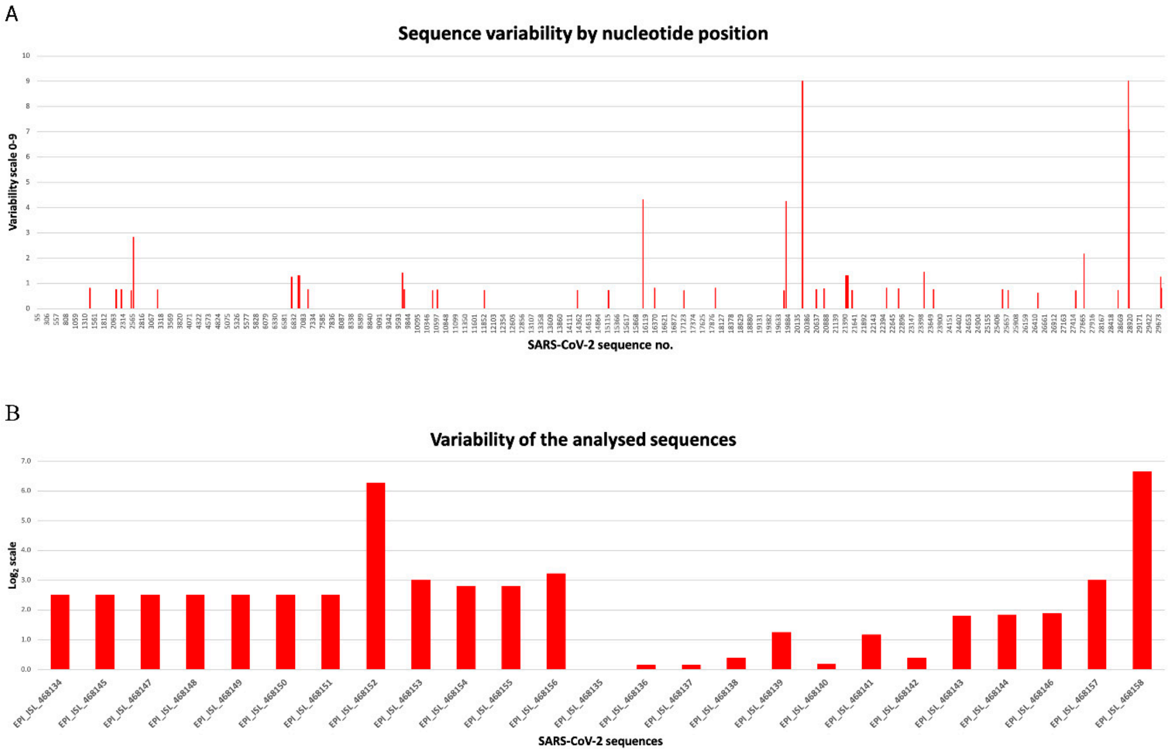

Supplementary Figure S1. Variability analyses of generated sequences expressed as (**A**) Variability for each nucleotide position calculated within generated sequences group on a scale from 0 to 9 (where 9 is the highest variability in the group) and (**B**) Degree of variability for each sample in the study group expressed using a logarithmic scale.

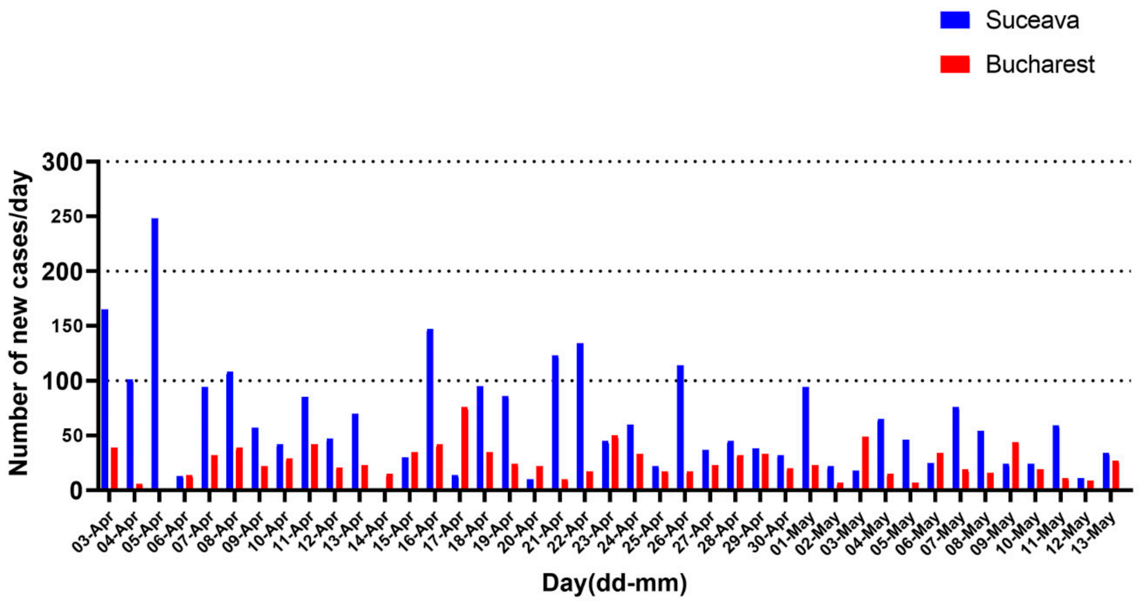

Supplementary Figure S2. The dynamics of newly reported SARS-CoV-2 cases in Bucharest and Suceava over a two-month period (April−May 2020).

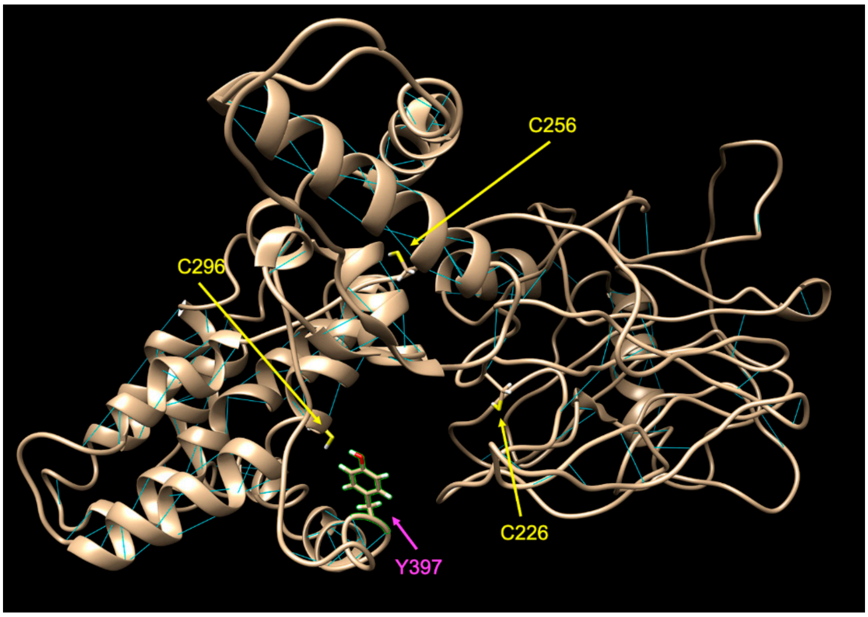

Supplementary Figure S3. C-I-Tasser model of Nsp4 - local representation of tertiary structure (using Chimera) around Y397C mutation. The possible effect of Y397C mutation in Nsp4: the aminoacids that may lead to the formation of disulfide bridges are shown as sidechains in the Nsp4 model.

© 2020 by the authors. Licensee MDPI, Basel, Switzerland. This article is an open access article distributed under the terms and conditions of the Creative Commons Attribution (CC BY) license (http://creativecommons.org/licenses/by/4.0/).
